# Modelling the Complexity of Human Skin In Vitro

**DOI:** 10.3390/biomedicines11030794

**Published:** 2023-03-06

**Authors:** Elisabeth Hofmann, Anna Schwarz, Julia Fink, Lars-Peter Kamolz, Petra Kotzbeck

**Affiliations:** 1 COREMED—Centre of Regenerative and Precision Medicine, JOANNEUM RESEARCH Forschungsgesellschaft, 8010 Graz, Austria; 2 Division of Plastic, Aesthetic and Reconstructive Surgery, Department of Surgery, Medical University of Graz, 8036 Graz, Austria; 3 Research Unit for Tissue Regeneration, Repair and Reconstruction, Division of Plastic, Aesthetic and Reconstructive Surgery, Department of Surgery, Medical University of Graz, 8036 Graz, Austria

**Keywords:** in vitro skin models, reconstructed human epidermis, human skin equivalent

## Abstract

The skin serves as an important barrier protecting the body from physical, chemical and pathogenic hazards as well as regulating the bi-directional transport of water, ions and nutrients. In order to improve the knowledge on skin structure and function as well as on skin diseases, animal experiments are often employed, but anatomical as well as physiological interspecies differences may result in poor translatability of animal-based data to the clinical situation. In vitro models, such as human reconstructed epidermis or full skin equivalents, are valuable alternatives to animal experiments. Enormous advances have been achieved in establishing skin models of increasing complexity in the past. In this review, human skin structures are described as well as the fast evolving technologies developed to reconstruct the complexity of human skin structures in vitro.

## 1. Introduction

The skin is at the frontline of our body and functions as a cover that protects us from the often-harmful environment. Animal experiments were and still are commonly used to elucidate the basic structure and function of the human skin as well as the mechanisms behind wound healing and skin diseases. However, anatomical inter-species differences often compromise the translatability of animal based studies [1,2,3]. For example, mice are the most commonly used laboratory animals despite the fact that murine skin has more skin appendages, fewer epidermal layers and is only loosely attached to the underlying muscle. Indeed, pharmaceutical absorption studies performed on mouse skin do not always reliably predict the outcome on human skin [2,4]. Porcine skin morphology and physiology as well as wound healing processes are more similar to human skin and several porcine models for wound healing have been established. However, there are also differences between these porcine models and the human situation, which may affect translatability of results [5,6,7]. Modelling human skin in vitro would ensure translatability of results and reduce animal experiments during preclinical evaluation of novel therapy approaches. The “3Rs” (Replacement, Reduction and Refinement) principle of humane animal research is globally accepted and is embedded in many national and international legislations [3]. Regulation acts such as the 2013 European ban on animal testing for cosmetic products have led to an advance of in vitro methods to test efficacy and safety of dermatological products [8,9]. In this review, we will discuss human skin structures, how these skin structures can be reconstructed in vitro and what is still missing to model human skin in its full complexity.

## 2. Skin structure

**Figure 1:**
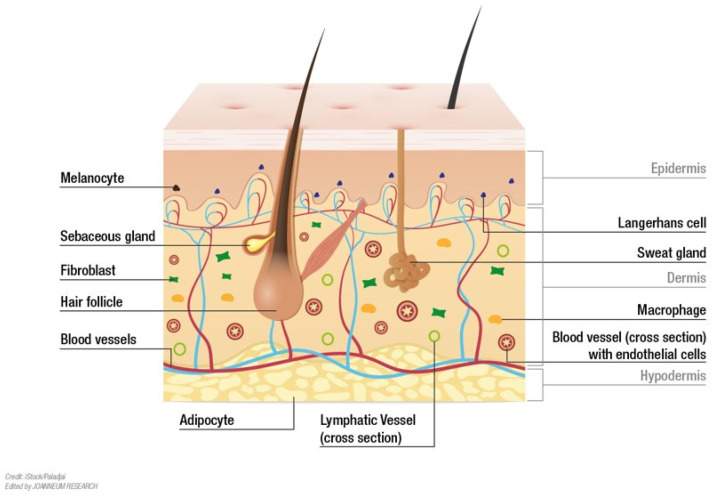
Structural details of human skin. The skin is composed of three distinct layers: the epidermis, dermis and hypodermis. The epidermis provides a barrier to pathogen invasion and regulates the amount of water released from the body. The dermis is tightly connected to the epidermis by the basement membrane; the dermis primarily consists of extracellular matrix, which is produced by fibroblasts. The dermis can be separated into two distinct layers, the superficial layer adjacent to the epidermis (papillary dermis) and a thicker layer below (reticular dermis). It also contains mechanoreceptors, thermoreceptors, hair follicles, sweat glands, sebaceous glands, lymphatic vessels, nerves and blood vessels. Those blood vessels provide nutrients and waste removal for both dermal and epidermal compartment [10].

The human skin is composed of diverse tissues that work together as a single structure to maintain internal body conditions (homeostasis) and that functions equally as a communicator to and a defense against the outside world. Skin is a constantly changing, dynamic organ that is involved in numerous processes vital to our health, e.g., regulation of the body temperature, balance of fluids, sensory reception, synthesis of vitamins and hormones. Human skin is composed of three distinct compartments, epidermis, dermis and hypodermis (Figure 1).

### 2.1. Epidermis

**Figure 2:**
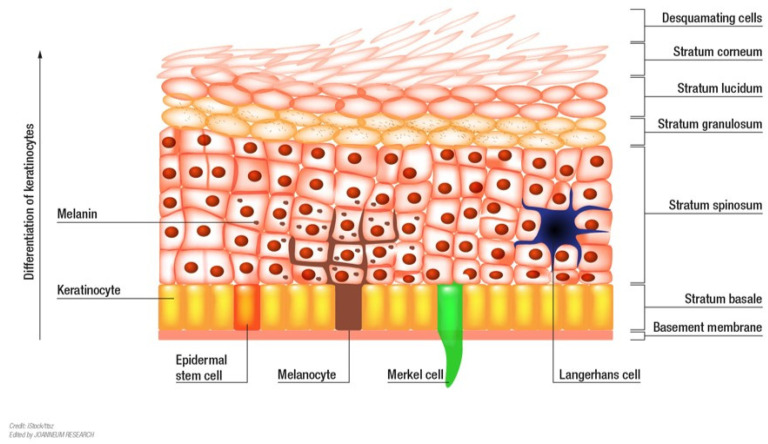
Epidermal structure in detail. The epidermis is a stratified squamous epithelium composed of multiple layers of keratinocytes. Basal keratinocytes proliferate and give rise to suprabasal successor cells that undergo a tightly controlled differentiation program to form distinct epidermal layers. In the stratum spinosum keratinocytes are already post-mitotic. Granular keratinocytes are terminally differentiated keratinocytes expressing loricrin, filaggrin and transglutaminase. Keratohyalin granules contain filaggrin and regulate hydration and cross-linking of keratin filaments. Upon differentiating to corneocytes, granular keratinocytes produce and secrete lamellar bodies, containing lipids and proteins, to the extracellular space, resulting in formation of hydrophobic cornified envelope. Corneocytes are non-living cells that form the outermost layer, which is responsible for the water impermeability of the skin. Apart from keratinocytes, which represent the major cell type in the epidermis, other cell types including melanocytes, Merkel cells and Langerhans cells are also present [15,16].

The epidermis is the topmost layer that interacts with the environment [10]. It creates an effective barrier against toxins, pathogens and dehydration [11]. The epidermal thickness ranges between paper-thin (30 µm on the eyelids) and up to 600 µm on areas that need to be particularly robust, e.g., palms and soles [12,13,14]. The epidermis is constituted of five sublayers (strata) of tightly interconnected building blocks, the keratinocytes (Figure 2).

Keratinocytes originate from epidermal stem cells that are anchored to the basal membrane of the innermost skin layer, the “stratum basale”. Here, the epidermal stem cells constantly produce transit-amplifying (TA) daughter cells that rapidly divide until they differentiate and stop proliferation [15]. During this maturation process, TA cells migrate towards the skin surface and undergo gradual changes in terms of appearance, organelle structure and cytoskeletal organization that result in the formation of sublayers that are distinct in regards of appearance and function [16]. Keratinocytes at different stages of maturation produce distinct types of keratins that provide structural support and play an important role in cell viability and signaling pathways associated with protein synthesis, cell growth and cell differentiation. Basal keratinocytes predominantly express keratins 5 and 14 filaments that tightly anchor keratinocytes to the basement membrane [17,18]. In the course of maturation, expression of keratins 5 and 14 is down regulated and eventually replaced by expression of keratins 1 and 10. Additionally, the expression of further structural proteins such as involucrin, profilaggrin, loricrin and trichohyalin along the maturation process [19]. Finally, keratinocytes have differentiated to corneocytes, which are cells without a nucleus, tightly packed with keratins 1 and 10, surrounded by a tough polymer structure termed the cornified cell envelope, which is composed of a protein and a lipid component. The protein part is made of involucrin and loricrin, which are cross-linked by transglutaminase enzymes, small proline rich proteins, late envelope proteins and filaggrin [20,21]. The lipid envelope consists of ceramides ω-acylated-hydroxy-ceramides and omega-hydroxy-fatty acids that are covalently attached to the protein layer [22]. Altogether, a hydrophilic barrier is formed that prevents fluid loss and protection against pathogens [23].

In addition to keratinocytes, the epidermis contains other cell populations. Melanocytes are found in the basal layer and are responsible for the production of melanin, a skin pigment that absorbs UV-radiation and that determines the skin color [24]. Merkel cells are located in or near the basal layer and act as sensory receptors in the skin [25]. Langerhans cells are antigen presenting immune cells of the epidermis that are most prominent in the stratum spinosum. Similar to dermal dendritic cells but unlike tissue macrophages, they migrate to lymph nodes following antigen uptake, where they initiate cytotoxic T-cell immunity [26,27,28,29,30]. The epidermis is devoid of blood or lymph vessels and therefore depends on diffusion from the underlying skin layer, the dermis, for oxygenation, nutrients and waste removal [31].

### 2.2. Basement Membrane

The exchange of oxygen, nutrients and waste molecules is controlled bi-directionally by a semipermeable sheet of extra cellular matrix proteins (ECM), the basement membrane. This basement membrane compartmentalizes the skin, yet holds epidermis and dermis together [32]. Epidermal keratinocytes feature stud like protrusions, hemidesmosomes that integrate the intracytoplasmic keratin network inside the keratinocytes to a fine network of laminins in the epidermis-facing side of the basement membrane (lamina lucida) [33]. Below lies the lamina densa, a tight network of collagen IV fibers and laminins, cross-linked mainly with the proteoglycan perlecan and different isoforms of the glycoproteins nidogen for stabilization [34,35,36,37]. The sublamina densa tethers the lamina densa to the papillary dermis through anchoring fibrils, thick loop structures of collagen VII associated with collagen I and III [38,39,40]. In the dermal side, the loop endings fan out to form “anchoring plaques” that contain also collagen IV [40]. Some of the basement membrane proteins are secreted by both epidermal keratinocytes and dermal fibroblasts (Collagen subtypes IV and VII, perlecan), while nidogens solely stem from fibroblasts and laminins from keratinocytes [35,41,42].

### 2.3. Dermis

The thickness of the dermis depends on its location and may vary between 2 mm and 6 mm [43,44]. Its primary role is thermoregulation through regulation of the blood supply and aspiration, to provide oxygen rich blood to the epidermis and to remove epidermal waste products [45]. This elastic yet firm middle layer stores a large proportion of the body’s water and cushions the body against injury. The dermis is more heterogeneous than the epidermis and resembles a complex network comprising connective tissue, blood vessels, nerve endings, hair follicles, and glands. Dermal fibroblasts, which make up the major cell type in the dermis, secrete proteins of the ECM into the intercellular space: collagen and elastin give the skin strength and flexibility, while proteoglycans such as hyaluronic acid contribute to tissue hydration and viscosity [46]. Fibronectin plays a crucial role in wound healing and is involved in cell adhesion, growth, migration, and cell differentiation [47]. Most of the skins immune competent cells are found in the dermis, which contains dermal dendritic cells, macrophages, mast cells, eosinophils, neutrophils, B-lymphocytes and T-lymphocytes such as natural killer T cells, αβ- and γδ-T-cells. These cells play an important role in the prevention of infections and the reconstruction of damaged tissue [18,48,49,50,51,52].

The dermis is further divided into two sub-layers: The layer closest to the epidermis is the stratum papillare. It has a spongy structure that is composed of loosely woven collagen fiber bundles, ECM and connective tissue. It is extensively vascularized and forms finger-like projections that protrude into the epidermis for increased exchange with the dermis. If these unique structures are pronounced, i.e., at the palms of the hand or the sole of the foot, they give rise to what is known as “fingerprints”. The stratum papillare contains many nerve endings responsible for the sensory transmission of touch, vibration or heat [45]. The lower layer, the stratum reticulare, consists of strongly interconnected elastic and collagen fibers that give the dermis its properties of firmness, extensibility and elasticity. It is home to hair roots, lymphatic- and blood vessels, nerves, sebaceous (oil secreting) and sweat glands [18,53].

### 2.4. Hypodermis

The hypodermis, also known as the subcutis or subcutaneous layer, anchors the dermis to the underlying muscles and bones. It is a well-vascularized, loosely textured tissue that contains larger nerves and blood vessels, connective tissue and, most importantly, white adipose tissue [44]. Besides fibroblasts, the hypodermis harbors adipocytes and the so-called stromal-vascular cell fraction consisting of mesenchymal stem cells “preadipocytes”, endothelial cells, pericytes, T cells, and macrophages [54,55]. The fat is stored in the form of large lipid droplets inside mature adipocytes and functions as an energy reserve for the body and provides insulation against cold or heat and physical protection [56]. Moreover, it performs as an endocrine organ that secretes a wide range of hormones involved in the regulation of dietary intake, glucose homeostasis, stimulation of angiogenesis, inflammation and hair growth [57].

A fully functional, fully human in vitro model should provide native skin structure and functionality; it should contain many different cell types that are able to proliferate and differentiate, as well as adipose tissue, vasculature and appendages. This optimal model would feature immune cell infiltration upon stimulation as well as long-term culture.

## 3. Reconstructed Human Epidermis

**Figure 3:**
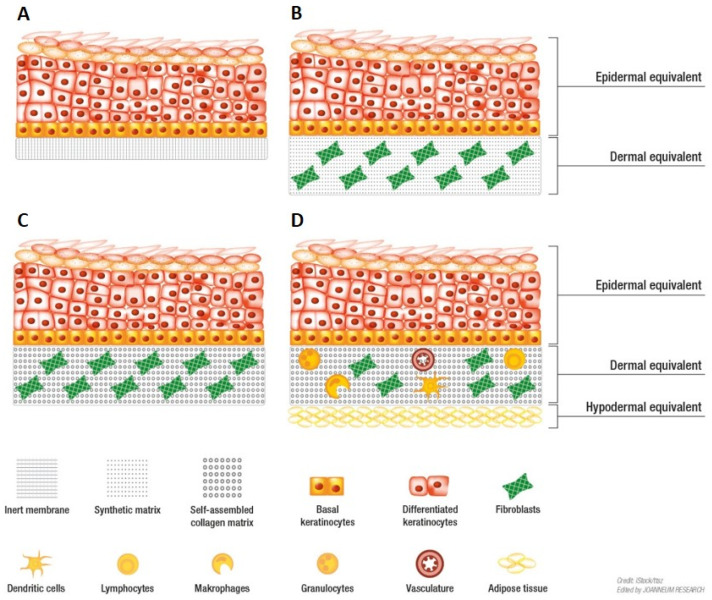
Evolution of human skin equivalents from reconstructed human epidermis (RHE) towards a fully functional, fully human skin model in vitro. (**A**) RHEs are generated by seeding and culturing keratinocytes on an inert matrix. Air-liquid-interface(ALI)-induced differentiation gives rise to a stratified epithelium that resembles a physiological epidermis [58,59]. (**B**,**C**) Reconstructed human skin (RHS) models feature a dermal equivalent where fibroblasts are grown in a synthetic matrix (**B**) [64,65,66]. Alternatively, fibroblasts can be induced to produce their own “natural” ECM (**C**) [67]. Keratinocytes are then seeded on top of the dermal equivalent and keratinocyte differentiation is induced by ALI. (**D**) An envisioned human skin model mimicking native human skin would consist of three layers, i.e., an epidermal, dermal and hypodermal equivalent. Moreover, a functional circulation, immune and nervous system would be of great interest.

For reconstructed human epidermis (RHE), normal human keratinocytes are isolated from juvenile foreskin or adult abdominal skin and expanded in culture medium. The cells are then seeded into transwells and shortly cultivated under submerged conditions before cultures are raised to the air-liquid-interface, which triggers keratinocyte differentiation (Figure 3A) [58,59]. In a continuous process lasting for about two weeks an epidermis-like structure is formed, which has been shown to reliably reproduce the morphological (well-stratified epithelium with basal, spinous, granular and corneal layers), biochemical and physiological properties of the human epidermis [60,61,62]. In a whole genome microarray study Gazel et al. showed, that the transcriptomes of human skin and RHE differed especially in genes known to be dependent on additional cell types and the crosstalk between keratinocytes and these additional cell types, such as fibroblasts. Human skin and RHE overlapped in critical epidermal differentiation markers, including keratins K1, K10 and K2e [63].

RHEs, some of which may be implemented in validated test procedures according to the EU Reference Laboratory for alternatives to animal testing (EURL-ECVAM) or the Organization for Economic Co-operation and Development (OECD), are commercially available for hazard assessments of the substances in accordance to the European Union regulation guidelines for registration, evaluation, authorization and restriction of chemicals (REACH) and can be obtained from a number of companies (Table 1). For quality assurance and standardization, the companies provide detailed information on cell types, cell handling, type of the construct, histology, immunohistochemistry, lipid analysis, as well as on permeability for standard substances and resistance to irritants [68]. In addition to these commercially available models, efforts have been made to also validate an open source RHE for in vitro toxicology testing [69,70,71]. Also pigmented RHE, which contains melanocytes of different phototypes in the basal layer of the epidermal construct, can be produced [72] or purchased. These model systems are used to analyze phototoxicity, or to test the effectiveness of commercially available sunscreens in a highly standardized manner [73,74].

## 4. Human Skin Equivalents

In addition to a fully differentiated epidermal layer, human skin equivalents (HSEs), also termed full thickness skin models or reconstructed human skin, include a dermal layer, which in the basic variant contains dermal fibroblasts seeded in a 3D matrix (Figure 3B). The general protocol to build HSEs includes the preparation of a fibroblast-loaded dermal scaffold, which is cultured for a few days to a week. This is followed by keratinocyte-seeding on top of the dermal scaffold. Similar to the production of RHEs, the HSEs are incubated under submerged culture conditions for a short time, before the air-lift is performed and keratinocyte differentiation is initiated [64]. Some standardized HSEs are also commercially available from different companies (Table 1). Fibroblasts were shown to positively influence keratinocyte proliferation and differentiation [65,66]. Moreover, paracrine signaling between fibroblasts and keratinocytes was shown to be essential for a well-structured basement membrane [75,76].

For dermis reconstruction, several scaffold compounds have been employed so far, with collagen type I being the most widely used, because it is the major component of the dermal ECM, and it is easily isolated from natural sources [77]. However, collagen type I gels exhibit weak mechanical properties and are susceptible to contraction exerted by fibroblasts [78]. Efforts to improve the stability of the scaffold matrices include chemical cross-linking, non-enzymatic glycation and more recently the combination of two or more natural polymers [79]. For example, a cross-linked combination of silk and collagen has been reported to perform in an excellent way, as it provides the cell-binding domains of the collagen, while benefitting from the superior mechanical characteristics of silk [80,81,82]. Synthetic polymers have been tested for their use as dermal scaffolds based on their adjustable physical properties, such as porosity and elasticity. Cells typically adhere rather poorly to these synthetic polymers, therefore they are usually employed in combination with natural polymers, which seem to be crucial for the physiological character of a reliable in vitro skin model [79]. Peptide-based hydrogels are another promising approach for tissue engineering HSEs [83,84]. Moreover, a method of a “self-assembled skin equivalent” has been described, providing an approach without any artificial scaffold components [67] (Figure 3C). Here, fibroblasts were grown for five weeks in the presence of ascorbate and produced thick fibrous sheets composed of stacked fibroblasts and exhibited an ultrastructural composition similar to normal connective tissue.

In order to imitate nature as closely as possible, the “basic” full thickness skin model can be equipped with additional cell types and structures including vascular, immune and nerve cells, as well as adipose tissue (Figure 3D).

### 4.1. Immunocompetence

Immunocompetence is highly desirable for a physiological in vitro human skin equivalent to be able to investigate processes dependent on immune cell functions such as wound healing, defense against infections, inflammatory conditions or allergic reactions [85,86]. Several approaches including the incorporation of T-cells, dendritic cells and/or macrophages of varying origins have been applied [87,88,89,90,91]. One major limitation of these approaches is the cell specific need for different nutrients and media composition. Media necessary for dermis/epidermis differentiation were shown to affect immune cell survival and function [92]. Another limitation is the lack of immune cell flow due to the lack of appropriate vascularization of HSEs [85,86].

### 4.2. Vascularization

Vasculature is essential for homeostasis and inflammatory responses, because it provides oxygen and nutrients and allows immune cell infiltration during inflammation. Approaches to mimic vascularization of the human skin equivalents use endothelial cells seeded within the dermal compartment for tube formation. It was shown that the origin of the endothelial cells as well as the dermal matrix composition are critical factors for optimal tube formation [93,94]. Experiments on pre-vascularization of dermal substitutes with adipose tissue-derived microvascular fragments yielded promising results, as increased density of microvascular and lymphatic networks was observed [95,96]. Groeber et al. were the first to introduce a vascularization approach that allowed true perfusion of the vessels with physiological pressure as well as bi-directional transport of molecules [70,97]. Bioprinted vascular-like structures were populated with human endothelial cells or endothelial cells derived from induced pluripotent stem cells (iPSCs), which resulted in a perfused skin equivalent, that could be used to study drug delivery [98]. In general, technologies such as microfluidics and bioprinting [99] in combination with innovative scaffold materials and iPSC-technologies are promising tools for the development of vascularized skin equivalents as well as for skin (body)-on-a-chip approaches.

### 4.3. Nervous System

In order to generate innervated skin equivalents, dorsal root ganglia neurons of either porcine [100] or murine origin [101] were integrated in otherwise fully human skin equivalents. Incorporation of neurons resulted in the production of the neuropeptides substance P and calcitonin gene-related peptide [100], as well as in accelerated wound healing [101]. Induced human neural stem cells (ihNSCs) can be generated by direct reprogramming of dermal fibroblasts [102]. These ihNSCs differentiate into neurons independent of media composition, they are robust and they maintain their neural phenotype even in complex co-cultures. Using the ihNSC approach a fully human innervated HSE could be successfully produced [80]. The addition of nerve cells resulted in donor specific cytokine secretion profiles arguing for the importance of the cross talk between skin cells, adipocytes, immune cells and nerve cells to reflect the in vivo situation [80].

### 4.4. Adipose Tissue

Surgeries, burns, chronic wounds or injuries affect underlying skin areas and therefore require reconstruction of the hypodermis to restore structural features and function [103]. Up to date, clinicians use skin grafts whose success can be limited by donor site availability or donor site morbidity [104,105]. Artificial skin equivalents with a functional subcutaneous layer, sourced from subcutaneous fat, could overcome these limitations. This concept is very attractive considering the fact that subcutaneous fat is abundant and readily accessible through the minimally invasive method, liposuction. Current commercial skin models do not include adipose tissue, but recent engineering approaches aim for the incorporation of adipose tissue cells in order to achieve a physiological skin anatomy and execution of vital functions such as hormone secretion [81,106,107]. Moreover, tri-layered skin models would have great potential for the application as advanced screening systems in the cosmetic and pharmaceutical industry because they mimic the natural situation better than bi-layered models [81,106,107].

Most models employ adipose-derived stem cells (ASCs) because they are easy to isolate from adipose tissue, resistant to mechanical stress and easy to increase in vitro [108]. For the composition of adipose tissue, these cells need to be differentiated into mature adipocytes through a treatment with drug cocktails, which is very time-, material-, and cost-intensive [109,110,111]. Another major drawback of ASCs is the lack of available culture media that allow adipogenic differentiation and concurrent epithelization of keratinocytes [111]. Common strategies are similar to the technique described by Monfort et al.: a culture of ASCs is embedded in a hydrogel matrix and cultivated under differentiation conditions. Depending on the rate of differentiation efficiency, the resulting hypodermis contains mature adipocytes as well as undifferentiated cells. The hypodermis is overlaid with a dermal equivalent that contains fibroblasts. Finally, keratinocytes are seeded on the surface, followed by cultivation at the air-liquid interface for the formation of an epidermis [107].

Some methods use mature adipocytes to overcome this problem. They can be isolated in high quantities from adipose tissue and used immediately without the need of a differentiation step. Major limitations are the fragility of mature adipocytes and different media requirements of adipocytes, fibroblasts and keratinocytes [111,112]. As a result, adipocytes tend to de-differentiate under suboptimal culture conditions. Huber et al. successfully co-cultivated adult adipocytes, fibroblasts and keratinocytes in a photo-crosslinkable methacrylated gelatin 3D matrix using a single commercial growth medium [113]. The generated tri-layered skin construct displayed a morphology similar to native skin tissue, but it disintegrated after 2 weeks cultivation time, possibly due to missing vascularization in the hypodermis layer. In order to maintain a high concentration of adipocytes in the hypodermis layer, Kober et al. followed a combined approach in which they embedded both mature adipocytes and ASCs. As scaffold, they used fibrin because of its biodegradability, biocompatibility and excellent mechanical properties that reproduce the soft character of adipose tissue. The final construct was cultivated for further 3 weeks in which all cell types stayed proliferative and ASCs were able to differentiate into mature adipocytes [114].

Other models are based on the inclusion of native adipose tissue. This method has the advantage that cell types other than adipocytes, such as ASCs, endothelial cells, smooth muscle cells, and macrophages, are introduced into the HSE. For example, Atac et al. added native adipose tissue to a dermis/epidermis model, which was cultivated in a perfused chip-based bioreactor. They were able to decrease cell apoptosis in the adipose tissue, resulting in increased keratinocyte proliferation in the basal layer of the epidermis [115]. Vidal et al. showed that the use of liposuction material introduces immunocompetence, as their HSE were capable to secrete increased levels of pro-inflammatory cytokines. This observation marks an important step towards the development of in vitro tissue systems that can be used for studying chronic skin diseases [80].

### 4.5. Skin Appendages

Skin appendages, i.e., hair follicles, sebaceous glands and sweat glands, are essential for various skin functions, including barrier function and thermoregulation. Hair follicles are of special interest, as they harbour a stem cell niche and play an important role in wound healing [116]. Several approaches were successful in inducing the formation of hair follicle-like structures in vitro [117,118,119,120]. However, the 3D arrangement within an HSE still is a major issue [121]. Recently, a combinatory approach of reprogrammed dermal papilla cells and 3D printed scaffolds was used to produce a fully human HSE with intact hair follicles [122].

### 4.6. 3D Bioprinting of Skin Constructs

3D bioprinting is a fast evolving technology of producing three-dimensional complex biological structures using living cells, biomaterials (bioinks), and biological molecules as input material for layer-by-layer printing. In this additive manufacturing process, custom-designed tissue constructs can be generated in a highly flexible and reproducible manner [123,124]. Using computer-aided design approaches, complex, heterocellular structures are produced with anatomical precision providing control over important parameters e.g., pore size, crosslinking and density of ECM. The first bioprinted skin equivalents were described back in 2009, where skin cells [125] or skin cells together with mesenchymal stem cells [126] were printed into a collagen matrix. Since then tremendous advances have been reported in the field of 3D bioprinting technologies in general and 3D bioprinting of skin equivalents. Several recent reviews give very detailed insight into the role of different printing technologies, as well as into the use of different structural components, i.e., biomaterials or bioinks, and various cell types [99,123,127,128].

Three main techniques are known to be used for 3D bioprinting. Pressure-based bioprinting, or extrusion bioprinting, is the most popular technique for skin tissue engineering as it allows a wide range of biomaterials with different viscosities being printed at room temperature. The technique of pressure-based bioprinting applies pneumatic pressure or mechanical pistons for continuous deposition of the biomaterials [127,128,129]. Inkjet-based bioprinting uses a drop-on-demand printing mode usually mediated by thermal or piezoelectric effects [130]. The biomaterial for inkjet-based bioprinting should have low viscosities, which will have an effect on the mechanical properties of final scaffolds [124]. Both printing technologies, pressure- and inkjet-based bioprinting, involve the ejection of the biomaterial through a tiny nozzle, which has been described to clog easily [126]. In order to avoid clogging as well as shear-stress-mediated cell damage, laser-assisted bioprinting can be used. Laser-assisted bioprinting provides a resolution of nearly a single cell (about 20 µm), which allows high cell densities to be printed [126,128]. Similar to the generation of HSEs, several natural or synthetic compounds are available for bioprinting skin as so-called biomaterials or bioinks. These biomaterials must be biocompatible, e.g., non-toxic and non-immunogenic, and they must be printable, which is dependent on a biomaterial’s rheological characteristics such as viscosity [131], gelation properties and on its capacity to be cross-linked [132,133]. Finally, these biomaterials must provide functionality by serving as a physical support as well as by influencing cell behaviour, such as proliferation and migration [128,134]. Finding the best balance between biocompatibility and mechanical characteristics is the most challenging part in 3D bioprinting. Biomaterials that provide good biocompatibility in terms of mimicking a tissue-like environment optimal for cell viability and functionality often lack the mechanical characteristics needed for printability and stability. In contrast, biomaterials providing optimal mechanical characteristics are often less well-suited for cell housing [128,134,135].

The choice of cells being printed into skin constructs was mainly driven by the two major skin cell types, i.e., fibroblasts and keratinocytes [127,128,136]. Several other skin cell types have also been used in 3D bioprinted skin, including melanocytes, endothelial cells, pericytes, microvascular endothelial cells follicle dermal papilla cells and also stem cells from various sources [136]. 3D bioprinting is a promising approach for the future production of skin constructs for research as well as for skin grafting. The possibility to produce anatomically precise structures including different cell types is a highlight of 3D bioprinting, especially regarding the option to include vasculature-like structures [137,138,139].

## 5. Limitations of Current In Vitro Skin Models

Despite the technological advances in (skin) tissue engineering there are still several tasks to tackle in order to model human skin in vitro mimicking all the structures and functions essential for skin research as well as for skin grafting. The majority of published HSEs show a simplified bi-layer-setup, i.e., epidermal and dermal compartment, populated with simplified cellular components, i.e., keratinocytes and fibroblasts, respectively. While this is sufficient to reproduce the dominant structures and functions, a more accurate micro-architecture as well as a more diverse cellular composition would be needed to capture subtle yet critical cell-cell, cell-matrix and dermal-epidermal signaling events.

Future HSEs would benefit from a tri-layer-setup including a hypodermal compartment, which would correspond to physiological skin anatomy and mediate important functions such as hormone secretion [81,106,107]. Liposuction material as a hypodermal equivalent was even shown to introduce—at least to some extent—immunocompetence to an HSE [80].

Introducing immunocompetence to HSEs is a major issue in tissue engineering, as the absence of immune cells limits the physiological relevance of current HSEs. Especially, inflammation, a crucial driver of wound healing, is dependent on tissue-resident immune cells as well as on immune cells recruited from the circulation [86]. An important challenge is to provide all necessary signals for the different immune cell populations whilst preserving keratinocyte, fibroblast and possibly adipocyte viability and functionality. For example, media composition was shown to be a critical factor when incorporating macrophages into an HSE [92].

Vascularization of the HSE allowing true perfusion of the skin construct is another major challenge on the way to a more physiological in vitro skin model. Perfusion of the dermal (and hypodermal) compartment would be essential for research on the development of skin diseases, wound healing, metastasis of malignant melanoma, tumor-angiogenesis, pathologies of the immune system, and pharmacodynamics. An HSE providing a vasculature based on a decellularized xenogenic matrix with conserved and perfusable vasculature structures [140] is a promising approach. 3D bioprinting advances show promising results [137] as well, although the dermal microcirculation cannot be fully imitated yet.

HSE culture approaches require incubation periods of 3–4 weeks or greater to form fully differentiated skin. The throughput available from these models is relatively low and, thus, a limiting factor for conducting high-throughput studies. 3D bioprinting is a fast evolving promising technology, which could be able to solve some of the issues described above. However, it needs expert personnel as well as cost-intensive equipment.

## 6. Conclusions

Complex HSEs are valuable and promising tools for basic research on skin structure and function. Innovative technologies such as the use of iPSCs and 3D bioprinting are of course demanding, but will have a lasting impact on human skin model evolution. In addition to basic research, future even more complex skin models may be used also for the research on skin diseases and cutaneous wound healing with a high translational efficacy. Standardized generation of human full thickness equivalents will allow for their use in drug screening experiments, thus replacing or at least reducing the need for animal experimentation.

## Figures and Tables

**Table 1:** Commercially available human skin models.

Type	Brand Name (Company)	Cell Types	Scaffold	Validated for
**RHE**	SkinEthic™ RHE (Episkin, Lyon, France)	NHEK	Polycarbonate filter	Skin irritation test (EC TMR B.46; OECD TGL 439)
	EpiSkin™ (Episkin, Lyon, France)	NHEK	Collagen	Skin irritation test (EU TMR 440/2008/EC; OECD TGL 439)
	EpiDerm™ (MatTek Corporation, Ashland, MA, USA)	NHEK	Collagen coated polycarbonate membrane	Skin Corrosion (OECD TGL 431), Skin Irritation (OECD TGL 439), Phototoxicity (OECD TGL 498)
	EpiCS^®^ (Phenion, Düsseldorf, Germany)	NHEK	Polycarbonate membrane	Skin Corrosion (OECD TGL 431), Skin Irritation (OECD TGL 439)
	ZenSkin RHE model (Zen-Bio, Inc., Durham, NC, USA)	NHEK	Polycarbonate filter	-
	LabCyte EPI-MODEL (J-TEC, Ltd., Osaka, Japan)	NHEK	Filter insert	-
	KeraSkin™ (Biosolution Co., Ltd., Seoul, Republic of Korea)	NHEK	Unknown	-
	Skin+™ (Sterlab, Saint Bernard, France)	NHEK	Unknown	-
**pigmented RHE**	SkinEthic™ (Episkin, Lyon, France)	NHEK; Melanocytes of different phototypes	Polycarbonate filter	-
	MelanoDerm™ (MatTek Corporation, Ashland, USA)	NHEK; Melanocytes of different phototypes	Filter insert	-
	EpiCS^®^-M (Phenion, RHPE, Düsseldorf, Germany)	NHEK; Melanocytes of different phototypes	Polycarbonate membrane	-
	MEL/001 (StratiCELL, Gembloux, Belgium)	NHEK; Melanocytes of different phototypes	Polycarbonate filter	-
	KeraSkin-M™ (Biosolution Co., Ltd., Seoul, South Korea)	NHEK; Melanocytes	Unknown	-
	RHEP (Sterlab, Saint Bernard, France)	NHEK; Melanocytes of different phototypes	Unknown	-
**HSE**	T-Skin™ (Episkin, Lyon, France)	NHEK; human fibroblasts	Dermal equivalent	-
	Full Thickness model (Sterlab, Saint Bernard, France)	NHEK; human fibroblast	Collagen	-
	EpiDermFT™ (MatTek Corporation, Ashland, USA)	NHEK; NHDF	Collagen	-
	Phenion^®^ FT (Henkel AG & Co. KgaA, Düsseldorf, Germany)	NHEK; human fibroblasts	ECM proteins	-

Abbreviations: RHE: reconstructed human epidermis; HSE: human skin equivalent; NHEK: normal human epidermal keratinocytes; NHDF: normal human dermal fibroblasts; source: neonatal foreskin; alternatively adult abdominal (breast) skin; ECM: extracellular matrix; TMR: test method regulation; TGL: test guideline.
